# Editorial: Post-translational modifications of extracellular signaling molecules and antigens in immune and inflammatory responses

**DOI:** 10.3389/fimmu.2022.1057531

**Published:** 2022-10-20

**Authors:** Inna S. Afonina, Loredana Frasca, Gaby Palmer

**Affiliations:** ^1^ Unit of Molecular Signal Transduction in Inflammation, Center for Inflammation Research, VIB, Ghent, Belgium; ^2^ Department of Biomedical Molecular Biology, Ghent University, Ghent, Belgium; ^3^ Istituto Superiore di Sanità, National Center for drug research and evaluation, Rome, Italy; ^4^ Division of Rheumatology, Department of Medicine, Faculty of Medicine, University of Geneva, Geneva, Switzerland; ^5^ Department of Pathology and Immunology, Faculty of Medicine, University of Geneva, Geneva, Switzerland

**Keywords:** post-translational modifications (PTM), cytokines, proteolysis, desialylation, citrullination, carbamoylation, inflammation, antigen presentation

Post-translational modifications (PTMs) exponentially increase the complexity of the human proteome and ensure that the right form of each protein is expressed in specific cells across our bodies at specific time points. Addition of functional groups or proteins, as well as proteolytic cleavage, regulate protein structure, activity, localization, immunogenicity and interaction with other proteins. Intracellular signal transduction heavily relies on a cascade of PTMs that ensure rapid message relay, mobilization of key enzymes and activation of appropriate cellular responses. However, also proteins with extracellular function are subjected to PTMs, including proteolysis, ROS-induced oxidative changes, carbamoylation and citrullination, which often occur at sites of inflammation. PTMs of extracellular immune mediators and antigens exert profound influences on all aspects of immunity.

In this Research Topic, we collated original articles and reviews illustrating current research and opinions pertaining to immunoregulatory roles of PTMs in extracellular signalling and highlighting the increasing interest in studying these PTMs in the context of host defense, immunopathology, and cancer immunotherapy.

Proteolytic processing is an efficient method to regulate the biological activity of target proteins. Proteases can efficiently inactivate and degrade substrate proteins or, on the contrary, unlock their bioactivity by removing inhibitory domains. For example, proteolytic maturation of IL-1 family members by extracellular proteases, derived from infiltrating immune cells at sites of inflammation, is considered as a safeguard mechanism ensuring that the full biological potential of these cytokines is only unleashed when required (reviewed in 1). Additionally, also proteases from invading pathogens and allergens can activate some IL-1 family cytokines (reviewed in 1, 2). In fact, it has been recently proposed that IL-1 family members serve as sensors of aberrant proteolytic activity (aptly named ‘Activity Recognition Receptors’), which is often indicative of infection or tissue damage (2). Frezza et al. demonstrate here that IL-1α and IL-36 cytokines are processed and activated by a variety of proteases derived from common allergens of plant, insect, fungal and bacterial origin, further cementing the idea that IL-1 family cytokines have evolved to recognize and signal the presence of exogenous proteases of various origin.

Desialylation of proteins is perhaps a lesser-known PTM, yet removal of terminal sialic acid residues from glycan chains can drastically affect protein folding and function, influence protein-protein interactions by unmasking or masking binding sites for molecular partners, and expose or cover proteolytic sites. Lillehoj et al. give an excellent overview of how desialylation of mucins, highly glycosylated mucus-forming proteins, can contribute to the development of various pathologies, in particular pulmonary fibrosis and autoimmune diseases. Conversely, the authors also highlight the protective role of mucin 1 desialylation by neuraminidase 1 against *Pseudomonas aeruginosa* lung infection and further discuss the role of the mucin-neuraminidase axis in infectious diseases.

Protein citrullination and homocitrullination, also known as carbamoylation, occur on the basic amino acids arginine and lysine respectively and lead to the loss of positive charge, which consequently affects electrostatic properties and conformation of the modified protein. This PTM has attracted a lot of attention since autoantibodies to (homo)citrullinated peptides were identified as the most specific biomarkers for rheumatoid arthritis (RA). It has been originally proposed that particular RA-associated variants of the major histocompatibility complex (MHC) class II molecule HLA-DRB1 contain a common sequence motif, the so-called shared epitope, that favors binding of citrullinated peptides and thus promotes presentation of citrullinated antigens to auto-reactive T cells (3). However, in a minireview article, Roudier et al. present an intriguing alternative ‘hapten-carrier model’ postulating that it is the citrullinating enzyme PAD4 that is recognized by T cells and acts as a carrier for citrullinated peptides (the haptens), thus facilitating the production of anti-citrullinated protein antibodies. Interestingly, a variety of proteins involved in the pathogenesis of RA are citrullinated. For example, Grillet et al. detected citrullinated forms of matrix metalloproteinase 9 in the synovial fluid of RA patients, although a causative link between PTMs of matrix degrading enzymes and RA pathogenesis remains to be established. In addition to RA, citrullination and carbamoylation are actively studied in the context of tumour biology. Amongst others, these PTMs have been shown to regulate cell death, differentiation, epithelial-to-mesenchymal transition and metastasis (4). Furthermore, citrullinated peptides are presented by antigen-presenting cells to CD4 T cells (5) and the idea of using citrullinated tumour antigens for immunotherapy has recently attracted a lot of interest. Cook et al. nicely show that immunization with a homocitrullinated vimentin peptide induces modification-specific CD4-mediated IFNγ responses in mice and has an anti-tumour effect in a mouse model of melanoma. Symonds et al. analysed MHC II ligands eluted from melanoma cells and discovered that while some citrullinated epitopes identified by peptide elution are indeed capable of inducing potent anti-tumour Th1 responses, others promote IL-10 regulatory responses instead. Furthermore, peptides with similar binding affinity to MHC II molecules have potential ability to compete within a combined vaccine prompting the need to carefully choose and characterize the composition of vaccines based on (homo)citrullinated peptides.

In conclusion, we believe that this Research Topic provides a representative snapshot of current questions pertaining to immunoregulatory effects of extracellular PTMs. The contributions included in this Topic highlight interesting new developments, and provide directions for future research in areas ranging from host defense in barrier tissues, to pathogenesis of autoimmune and chronic inflammatory diseases, and to tumour immunobiology, thus emphasizing the broad influence of extracellular PTMs on immune responses in general.

## Author contributions

IA wrote the first draft of the manuscript. All authors contributed to the manuscript, read, and approved the submitted version.

## Funding

IA holds a fundamental mandate of the Foundation against Cancer (code 365C06721) and is supported by a Grant from the Fund for Scientific Research Flanders (FWO) (code 3G086521), as well as by an Early Career Research Grant from the National Psoriasis Foundation (grant No. 849077). LF’s work is supported by the National Psoriasis Foundation and by a FOREUM research grant. GP’s work is supported by the Swiss National Science Foundation (grant 310030-188470), the Rheumasearch Foundation, and by a generous donor advised by Carigest SA. The funder was not involved in the study design, collection, analysis, interpretation of data, the writing of this article or the decision to submit it for publication.

## Conflict of interest

The authors declare that the research was conducted in the absence of any commercial or financial relationships that could be construed as a potential conflict of interest.

## Publisher’s note

All claims expressed in this article are solely those of the authors and do not necessarily represent those of their affiliated organizations, or those of the publisher, the editors and the reviewers. Any product that may be evaluated in this article, or claim that may be made by its manufacturer, is not guaranteed or endorsed by the publisher.
